# Sweat rate and sodium loss during work in the heat

**DOI:** 10.1186/1745-6673-3-4

**Published:** 2008-01-29

**Authors:** Graham P Bates, Veronica S Miller

**Affiliations:** 1 School of Public Health, Curtin University of Technology, Perth, Western Australia

## Abstract

**Objective:**

Significant and poorly documented electrolyte losses result from prolonged sweating. This study aimed to quantify likely sodium losses during work in heat.

**Methods:**

Male subjects exercised in an environmental chamber on two consecutive days in both winter and summer. Sweat collecting devices were attached to the upper arms and legs.

**Results:**

Sweat rates were higher and sodium concentrations were lower in the summer (acclimatised) than the winter (unacclimatised) trials. Sweat sodium concentration was reduced on the second day in summer but not winter. Regional differences were found in both seasons.

**Conclusion:**

The difference between days in summer probably reflects short-term acclimation. The difference between seasons reflects acclimatisation. The data predict average sodium (Na) losses over a work shift of 4.8–6 g, equivalent to 10–15 g salt (NaCl). Losses are potentially greater in unacclimatised individuals.

Fluid and electrolyte losses resulting from prolonged sweating must be replaced to prevent imbalance in body fluids, however guidelines for this replacement are often conflicting.

This study provides important information for occupational health practitioners by quantifying the likely sodium losses over a work shift and providing recommendations for replacement.

## Background

During prolonged work periods in the heat (8–12 hour shifts), the maintenance of high sweat rates leads to progressive dehydration, which may be accompanied by impairment of mental and physical performance and of heat dissipation [1-4]. Dehydration will impair work capacity and may pose a serious risk to health [5]; the intake of fluid during the working period to replace sweat losses is therefore imperative.

However the sodium replacement need is often overlooked, mainly as a consequence of scant information regarding the sweat loss of sodium over time. There is also little information available concerning variability of sweat concentration from different regions of the body (is sweat sodium the same in all body regions) and between the same individual (unacclimatised and acclimatised). With a better understanding of electrolyte loss in sweat, accurate advice regarding replacement beverages can be provided to workers performing manual tasks in the heat.

Commercially prepared sports drinks have varying concentrations of glucose and sodium, and range from hypertonic to hypotonic with respect to plasma. Sodium is added to some drinks for the purpose of replacing sweat salt losses, and to aid in the transport of glucose across the intestinal wall. Glucose is added to the drinks in order to maintain blood glucose levels (avoid fatigue) during the work period. Sweat is hypotonic to plasma and to some of the electrolyte replacement drinks available. Consequently, the consumption of these electrolyte replacement drinks, if made available to workers ad libitum, may result in the consumption of too much sodium. On the other hand, if sweat losses are replaced with plain water a dilution of the plasma may occur to the point of the person being hyponatremic. It should be emphasized that sweat losses can exceed 1.5 litres/hour when working in very hot environmental conditions [6,7]. Meal breaks in order to allow salt and glucose intake from solid food are a must if workers are using water to replace sweat loss as nearly all food contains some sodium. However before appropriate sodium intake can be recommended, the loss over a work duration must be known.

Soft drinks and cordials have approximately 10% sugar content and if these are used as a sole replacement beverage this can significantly increase the daily kilojoule intake of the worker. During the summer when sweat rates are high, it is not uncommon for some workers to consume 10 litres of fluids in the working day. The daily sugar intake in this instance would be over 1.0 kg. In addition, cola and recently released "designer drinks" have a moderate to high concentration of caffeine. This can reduce fluid retention. Coffee and to a lesser extent tea are also caffeinated beverages, and large consumption (more than two cups per work shift) should be avoided especially during the summer when sweat rates can be high. Some drinks have a low pH (acidic) and high sugar concentration (10%), and while they may be appropriate for short duration sport sweat replacement, they should not be recommended for daily high volume consumption. Thus workers require education so that appropriate choices are made about replacement fluids. This is particularly true at the beginning of summer when they are unacclimatised to the heat; however we do not currently have a comprehensive understanding of sweat sodium losses in workers.

As sweat loss can be up to10–12 litres per day, and sweat contains sodium, an essential electrolyte, this study was designed to better understand sweat sodium loss so that informed educational strategies can be put in place in order to prevent heat illness and accidents due to the effects of heat strain in the workplace.

## Methods

The subjects were 29 healthy, male, manual outdoor workers (various trades) aged between 18 and 50 years, all provided informed consent to participation in the study. Typical summer temperatures in the study location would be 30–35°C, winter temperatures average 15–20°C. The cardiovascular fitness (mean VO_2_max), assessed using the Åstrand and Rodahl protocol was 33.7 mL.kg^-1^.min^-1 ^in summer and 39.1 mL.kg^-1^.min^-1 ^in winter. The subjects were assumed to be heat acclimatised during the summer experiments, and heat unacclimatised during the winter trials. One week following assessments, each subject performed two exercise-heat tests in a climate chamber on consecutive days in order to measure daily differences in sweat sodium. All heat tests were conducted in the morning. The climate chamber was maintained at 35°C and 50 % RH, air velocity was minimal, WBGT was approximately 29.3°C. TWL under these conditions for a subject wearing minimal clothing is approximately 180 W.m^-2^.

Before entering the climate chamber the subjects were weighed in minimal clothing on an electronic balance scale (accuracy ± 5 g), the subjects then changed into their exercise clothing (shorts and trainers)and their core temperature was recorded from the tympanic membrane (accuracy ± 0.1°C) using a common medical instrument (Braun).

Each subject was then fitted with a heart-rate monitor (Polar GBR 175015 A) and exercised on a cycle ergometer at 40 % of VO_2_max (equivalent to moderate manual labour e.g. mining or construction work) for a total of 35 minutes. The heart rate was recorded at 5-minute intervals throughout the testing session. There were no restrictions placed on the lifestyle of the subject prior to, or during, the testing period. The subjects were fitted with four sweat collecting devices after 15 mins of cycling, the time delay between exercise onset and attachment of the devices was to allow sweating to be initiated. This avoids any possible concentration changes between "start up sweat" and regular sweat flow. The collecting devices were Wescor sweat collection capsules [8] modified by extending the collection coil, and using custom made adjustable strapping to secure the capsule. Care was taken to ensure consistent, minimal pressure was applied to the skin. This was to avoid excessive pressure, yet prevent sweat leaking from the collection site. The capsules were positioned on the lateral aspect of both upper arms, and the front of both thighs, approximately midway between the knee and hip. The devices were secured to the limbs after the sites had been shaved and sterilised with alcohol swabs. The subjects continued to cycle for a further 20 minutes after the sweat collecting devices had been attached. Core temperature was monitored regularly. At the end of the exercise session, the sweat collecting devices were removed and placed in individual sealed plastic bags. The subjects were then instructed to shower without wetting their hair, abstain from drinking, eating, or urinating, and to ensure they were completely dry before re-dressing into the clothes in which they were originally weighed. After re-weighing, the sweat rate (mL. min^-1^) was calculated from the weight loss of the subject over time. The collected sweat was evacuated with compressed air, into small weighing trays. The sweat samples were weighed from each site for sweat rate comparisons, and then diluted in volumetric flasks with deionised water. The concentration of sodium was then determined by atomic absorption spectrophotometry.

Linear regression of data from contralateral sites (right and left) was carried out to confirm that differences did not arise from the methodology of either sweat collection or analysis. Probability of intra-individual variation between days, limbs, and seasons was analysed by student's paired t-test. Means and 95% confidence limits for group seasonal data were determined.

The experiments described in this paper were approved by the Curtin University Human Ethics Committee.

## Results

Sweat sodium concentrations from the relatively inactive arms were consistently higher than the active legs for both days in summer and winter as shown in Table 1. The mean sodium concentration in the 58 arm samples on the first day of sampling in winter was 72.7 mmol.L^-1^, and on the second day 72.9 mmol.L^-1^. Similarly, the sodium concentration in leg sweat did not significantly alter from day to day in winter (Table 1). However in the summer samples the sweat sodium concentration from both the arms and legs on day 2 showed a substantial reduction from day 1 samples as shown in the same table. The concentrations for the contralateral limbs for arms and legs of the same individual on the same day were virtually the same as shown by the correlation coefficients, (*r*) for each day between right and left arms and legs for each of the 29 subjects (Table 1). Analysis of the sweat sodium concentration data by paired t-test (Table 2) showed significant differences between arms and legs of individual subjects on both days and overall these differences are reflected in the means. In summer, the differences between days for the arms was significant and for the legs almost so, whereas in winter no differences were seen, correlating with the mean data in table 1.

**Table 1:** Sweat sodium concentration

		Mean day 1	***r*** day 1	Mean day 2	***r*** day 2
**Summer**	arms	53.3	0.853	44.3	0.983
	legs	42.9	0.958	38.5	0.981
**Winter**	arms	72.7	0.941	72.9	0.935
	legs	55.5	0.947	53.8	0.976

Mean values of sweat sodium concentration (mmol.L^-1^) from individual arms and legs taken on 2 consecutive days in summer and winter and correlation (***r***) between right and left limb data. (n = 29 subjects)

**Table 2:** Intra-individual variation.

		Summer **p **value	Winter **p **value
day 1, both arms	day 2, both arms	0.0002*	0.6774
day 1, both legs	day 2, both legs	0.0870	0.6437
day 1, both arms	day 1, both legs	0.0029*	0.0001*
day 2, both arms	day 2, both legs	0.0189*	0.0001*
both days, arms	both days, legs	0.0047*	0.0001*

Probability values from paired t-tests comparing sweat sodium data for each subject between days and between limbs. * Level of significance **p **< 0.05

**Figure 1:**
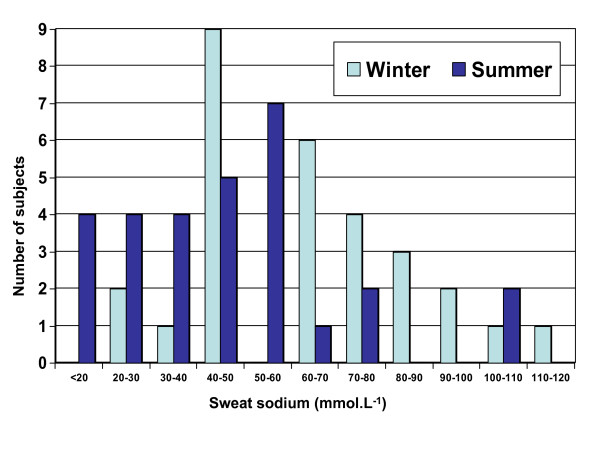
**Sweat sodium concentration (mmol.L-1) of 29 subjects participating in summer and winter heat tests**. The subjects worked at a set rate (40% VO_2_max) in a climate chamber set at 35°C and 50% RH for 35 mins. Values are means of samples from all anatomical sites.

The mean sodium concentration in sweat from both arms and legs showed a substantial difference between summer and winter (Table 3), as did the means of samples from all limbs (44.7 mmol.L^-1 ^in summer and 63.8 mmol.L^-1 ^in winter, p = 0.0001). The individual data for all limbs combined are presented graphically in Figure 1.

**Table 3:** Summary of mean seasonal data for sweat sodium concentration and sweat rate.

	Sweat sodium concentrations			Mean sweat rate (kg.h^-1)^
		
	Mean arms (mmol.L^-1^)	Mean legs (mmol.L^-1^)	Combined seasonal mean (all limbs) (mmol.L-1)	
Summer	48.4 ± 26.6	41.0 ± 23.3	44.7 ± 24.7	0.47 ± 0.14
	(38.1 – 58.7)	(31.3 – 50.6)	(35.7 – 53.7)	(0.29 – 0.65)
Winter	72.3 ± 24.9	55.5 ± 21.7	63.8 ± 22.6	0.41 ± 0.17
	(62.3 – 82.3)	(46.2 – 64.8)	(55.4 – 72.2)	(0.21 – 0.49)
**p**			0.0001*	0.0299*

Data are mean ± SD with 95% confidence limits (parentheses).

* Level of significance **p **< 0.05. Paired t-tests comparing individual summer and winter data.

**Figure 2:**
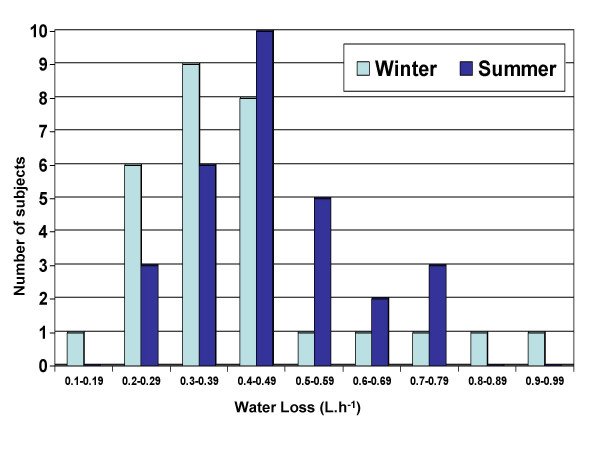
**Sweat loss (L.h-1) of 29 subjects participating in summer and winter heat tests**. The subjects worked at a set rate (40% VO2 max) in a climate chamber set at 35°C and 50% RH for 35 mins.

There was a significantly (p = 0.0299) greater sweat rate (water loss) in summer than in winter as shown in Fig 2. The mean water loss in the summer was 7.8 mL.min^-1 ^(0.47 L.h^-1^) compared with 6.9 mL.min^-1 ^(0.41 L.h^-1^) in winter (Table 3). Sweat rate ranged from a minimum of 0.1 L.hr^-1 ^to a maximum of 1.0 L.hr^-1 ^with a narrower range in summer than winter, both the minimum and maximum individual values of water loss were recorded in winter.

Regression analysis showed no significant correlation between subject body composition, fitness or age and either sweat sodium concentration or sweat rate.

## Discussion

Sweat sodium concentration collected from the right and left arms and legs on the same day showed a very strong correlation confirming methodological consistency [9]. However, a statistically significant intra-individual difference was demonstrated between sodium concentration in sweat secreted from the arms and legs, for both the summer and winter measurements (Table 2), also apparent in the mean data (Table 1). The sodium concentration in sweat samples taken from the legs was significantly less than from the arms (Tables 1&2). The difference in sweat sodium concentration between the arms and legs may be due to the difference in metabolic activity between the leg and arm muscles. The workload on the cycle ergometer required to reach 40 % VO_2_max is achieved by the leg muscles, producing significant metabolic heat energy, which has to be dissipated. However regardless of the causes of these regional differences the fact remains that sweat collection from one anatomical region may not be representative of whole body sodium loss.

There was a statistically significant change in sodium concentration between the first and second day in summer for the arms and to a lesser extent for the legs, suggesting that one heat exposure in summer is sufficient to trigger an acclimation effect. In winter this difference was not present. This short-term acclimation has previously been shown by Kirby and Convertino [10] however in their study sodium concentration was only measured on day 1 and day 10. As acclimation was being studied it is assumed the study was conducted in the cooler months. As the findings in the current study showed no variation in the first two days during winter, when subjects would be expected to be unacclimatised, it would appear that the triggering mechanism for increased sodium conservation in the unacclimatised state requires more than one heat exposure but is well established after 10 days. In contrast, in summer when subjects would be more acclimatised one exposure would appear to induce a sodium conservation response. The sweat glands may be more sensitive to aldosterone when in the acclimatised state. This was also postulated by Kirby and Convertino [10] who reported that decreased sweat sodium secretion was associated with significant reductions in plasma aldosterone during exercise in the heat following acclimation. The findings of the current study would reinforce increased sensitivity to aldosterone as the explanation for the seasonal differences. Further, the sensitivity is enhanced during summer when sodium retention would be important in order to prevent electrolyte disturbance due to chronic high sweat sodium loss.

The absence of any relationship between body composition, fitness or age and either sweat sodium concentration or sweat rate may come as a surprise, as exercise produces metabolic heat, which in turn induces sweating. On this basis it could be hypothesised that fitter individuals exercise more and therefore would have greater sodium conservation due to exercise-induced acclimatisation, however this appears not to be the case. Whether the metabolic heat generated is insufficient or environmental heat is a requirement remains to be fully demonstrated.

Sweat sodium concentrations in summer were less than in winter, the mean value for summer being 44.7 mmol.L^-1 ^and winter 63.8 mmol.L^-1^. However the standard deviations for summer and winter were similar (24.1 in summer and 22.6 mmol.L^-1 ^in winter). Therefore the variability in sweat sodium concentration was greater in summer than in winter (apparent in Figure 1). This supports variation reflecting inherent rather than lifestyle differences, as individuals seem to differ in their ability to acclimatise to the same environmental stress. The ethnicity of subjects was not recorded but genetics determining sweat gland density and sensitivity (receptors on the sweat duct) may have more influence than thought on the sweat response and the ability of the sweat gland to reabsorb sodium. Seasonal change to sodium loss reflects the well-known acclimatisation response. All the subjects were outdoor workers and were tested at the end of the summer months, when their acclimatisation would be expected to peak, and near the end of winter.

Future experiments should aim to clarify whether leg sweat glands have an inherently different capacity to sweat compared with the arms. Alternatively, since legs generally have a greater workload than arms, a training effect could occur to sweat glands in the lower limbs that results in a greater absorptive ability due to ductal hypertrophy or an increase in the concentration of enzymes involved in reabsorption, a possibility given some credibility by Fox et al [11] who showed a training effect on sweat glands. Similar findings were reported by Hofler [12].

One criticism of using local sweat collection methods has been that sodium concentration is usually higher than with using whole body techniques [13]. Shirreffs and Maughan, who measured sodium loss using whole body washdown, reported sweat sodium as 50.8 mmol.L^-1^. However the time of year the study was conducted is not stated, so whether the subjects were acclimatised is not known. The mean sweat sodium concentration in summer (44.7 mmol.L^-1^) for the current study was slightly less than that described by Shirreffs and Maughan [13], the winter value (63.8 mmol.L^-1^) was higher as would be expected if unacclimatised were to be compared to acclimatised subjects. There is sound agreement between the two methods.

From a practical viewpoint, a number of findings from this study can be put to use by occupational physicians. It is common for miners and other manual workers to perform 12-hour shifts in hot environments. The sweat loss can be as high as 12 litres per day [14] but 8–10 litres is common [6]. This represents a substantial fluid loss and demonstrates the importance of maintaining hydration status when working in the heat. These losses represent a substantial percentage of body weight and will rapidly lead to dehydration unless replacement fluid is consumed. In addition the sodium (Na) loss from sweating at this rate could exceed 10 g per day equivalent to 25 g of salt (NaCl). In this study the individual variation in both sweat rate and sodium concentration was substantial, however based on the mean data the sweat loss over a 10-hour shift even in a moderate environment would be 4.7 litres in summer and 4.1 litres in winter. There is currently no simple method to predict an individual's sweat composition, however on the basis of this study the average sodium concentration would be 45 mmol.L^-1 ^in summer and 64 mmol.L^-1 ^in winter (Table 3). The average acclimatised and unacclimatised sodium (Na) losses for a 10-hour shift in a moderate environment (35°C, 50 % RH) at 40% of VO_2_max would therefore be 4.8 g and 6 g, assuming the sweat rates and composition measured in this study were constant over the shift. The data predict that sodium loss would be greater in the unacclimatised individual (winter data) even with a lower sweat rate due to the higher sweat sodium concentration. Replacement of this daily electrolyte loss at regular intervals for individuals working in the heat is imperative in order to avoid possible electrolyte disturbance and impaired work performance. Given that the health message is to reduce sodium intake it becomes important that workers are educated as to the importance of eating during meal breaks and of having sodium rich foods when working in hostile conditions. Given the carbohydrate concentration in most sports drinks recommending these would not be sound; fluid replacement beverages should have far less carbohydrate and ideally more than 15 mmol.L^-1 ^of sodium, although in the authors' experience palatability limits sodium content.

## Conclusion

Based on the results of the current study the following conclusions and recommendations are provided:

1. People working in moderately hot conditions for 10 hrs on average will lose between 4.8 and 6 g of sodium (Na) equivalent to 12–15 g of salt (NaCl) depending on acclimatisation. However due to the substantial interindividual variation in sweat rate and sodium concentration individual losses may be much higher. This essential electrolyte must be replaced in order to avoid fluid imbalances, thus eating during the shift is a must.

2. One work session in the heat, for an acclimatised person is sufficient to activate sodium-conserving mechanisms. However in the unacclimatised worker longer exposure is required. A worker starting work in harsh conditions should be given 10 days or more to acclimatise before performing heavy manual work in the heat.

3. Cordials and sports drinks are contra-indicated for people working in hot environments due to the very high energy content. An ideal fluid replacement beverage for industrial use should have significant sodium content with minimum carbohydrate.

## Abbreviations

VO_2_max: Maximal oxygen uptake; RH: Relative humidity; WBGT: Wet bulb globe temperature; TWL: Thermal work limit.

## Authors' contributions

GB conceived the study and collected the majority of the data. VM collected some data. GB and VM analysed and interpreted the data and prepared the manuscript for publication. Both authors read and approved the final manuscript.
